# Defined Microbial Communities Modulate Polyphenol Transformation and Quality of Kombucha Across Different Tea Substrates

**DOI:** 10.3390/foods15111897

**Published:** 2026-05-28

**Authors:** Jiayi Zhang, Shengyang Shi, Yingxi Chen, Sufang Zhang, Chaofan Ji

**Affiliations:** State Key Laboratory of Marine Food Processing & Safety Control, National Engineering Research Center of Seafood, Collaborative Innovation Center of Seafood Deep Processing, School of Food Science and Technology, Dalian Polytechnic University, Dalian 116034, China; 241710832001008@xy.dlpu.edu.cn (J.Z.); 20171083200808@xy.dlpu.edu.cn (S.S.); chenyx@dlpu.edu.cn (Y.C.); zhangsf@dlpu.edu.cn (S.Z.)

**Keywords:** kombucha, defined microbial communities, tea substrates, phenolic transformation, antioxidant activity, sensory quality

## Abstract

Kombucha quality is largely governed by polyphenol transformation during fermentation. However, interaction between substrate composition and microbial communities regulating phenolic transformation and quality formation remains unclear. In this study, six tea substrates (white, green, yellow, black, oolong, and mint tea) were fermented using three defined microbial communities (SMC1-SMC3) and a traditional symbiotic culture of bacteria and yeast (SCOBY) to evaluate carbon metabolism, phenolic transformation, antioxidant activity, and sensory quality. After 10 d of fermentation, SMC2 and SMC3, containing acetic acid bacteria, showed stronger acidification (pH 2.2–2.5) and lower ethanol (0.34–0.52 mg/mL) than SMC1 (13.09–15.88 mg/mL). Phenolic transformation was substrate-dependent: total phenolics and flavonoids decreased in green tea, both increased in white tea, while flavonoids increased in oolong and black tea. Meanwhile, rutin decreased in white and green tea, whereas gallic acid accumulated in yellow, black, and oolong teas and was positively correlated with antioxidant activity. Sensory evaluation showed SMC3 achieved higher overall acceptability in most substrates, whereas SCOBY performed best in mint tea. These findings indicate substrate-microbiota interactions play a key role in phenolic transformation and quality formation in kombucha. Rational matching of tea substrates with defined microbial communities enables coordinated optimization of antioxidant activity, ethanol control, and sensory quality.

## 1. Introduction

Kombucha is a traditional beverage produced by the co-fermentation of yeasts and bacteria in sweetened tea broth [1,2]. Its distinctive flavor characteristics and potential health benefits have attracted growing interest. Among its bioactive components, polyphenols are particularly important: their transformation products serve as key contributors to antioxidant activity [3], while polyphenols themselves also directly affect bitterness, flavor complexity, and overall sensory quality. Therefore, polyphenol transformation during fermentation is regarded as a key process shaping both the functional characteristics and quality of kombucha. However, the relationships between polyphenol transformation and quality formation during kombucha fermentation remain insufficiently characterized, particularly under different substrate and microbial community conditions.

Traditional kombucha fermentation typically uses a symbiotic culture of bacteria and yeast (SCOBY) as the starter culture. However, the microbial composition and relative abundance within the SCOBY are usually not clearly defined, leading to insufficient reproducibility and unstable product quality [4]. This undefined microbial community structure also limits the reproducibility of polyphenol transformation and functional characteristics during fermentation. Consequently, the traditional SCOBY-based system limits the ability to establish a reproducible fermentation.

To improve controllability and product quality, replacing the traditional SCOBY with synthetic microbial communities (SMCs) of a defined composition has become a major research direction. Defined microbial communities have been shown to establish clearer links between microbial composition and metabolic performance, and their performance varies widely. For example, a three-species consortium, composed of *Acetobacter pasteurianus*, *Gluconacetobacter xylinus*, and *Zygosaccharomyces bailii*, increased total phenols, flavonoids, and gluconic acid while reducing ethanol, achieving a sensory quality comparable to that of a traditional SCOBY [5]. Other consortia have also demonstrated enhanced polyphenol transformation and antioxidant activity [6,7]. However, these studies mainly focused on microbial composition itself, while the interaction between defined microbial communities and different substrates remains poorly understood.

In addition to microbial communities, substrate composition critically affects kombucha fermentation and its final quality. In recent years, the substrates used in kombucha and kombucha-like beverages have extended beyond traditional tea bases to include non-traditional substrates such as fruit juices and herbs [8]. Differences in phenolic composition among these substrates may alter the microbial metabolic environment, thereby further influencing polyphenol transformation and quality. For example, fruit-based kombucha [9], herbal kombucha analogs (e.g., mint-based products) [10], and kombucha prepared from different tea types [11] have been reported to differ markedly in total phenolic contents, antioxidant activities, and sensory qualities. However, the combined influence of substrate composition and microbial community structure on polyphenol transformation and quality characteristics has not been systematically compared.

Therefore, this study employed six tea substrates, white tea (WT), green tea (GT), yellow tea (YT), black tea (BT), oolong tea (OT), and mint tea (MT) to systematically investigate kombucha fermentation using three defined synthetic microbial communities (SMC1-SMC3) and a traditional SCOBY. This study aimed to evaluate how different tea substrates and microbial community structures jointly influence carbon metabolism, polyphenol transformation, and quality characteristics during kombucha fermentation. By integrating analyses of microbial growth, metabolic characteristics, antioxidant activity, and sensory properties, this work seeks to clarify the matching relationships between tea substrates and microbial consortia, and to provide practical insights into the selection of suitable tea substrates and microbial communities for kombucha production.

## 2. Materials and Methods

### 2.1. Materials, Microbial Cultures, and Reagents

WT was purchased from Boqi Tea Trading Department of Panlong District, Kunming, China; GT from Xinyang Lantian Tea Co., Ltd. (Xinyang, China); YT from Hunan Pingyun Tea Co., Ltd. (Changsha, China); BT (Lapsang Souchong) from Hongzun Company (Quanzhou, China); OT from Fujian Yuanhangyuan Tea Co., Ltd. (Fuzhou, China); and MT from Zhongmin Piaoxiang Food Co., Ltd. (Xiamen, China). Sucrose was purchased from Meilin Company (Huludao, China).

*Acetobacter pasteurianus* (*A. pasteurianus*) CGMCC 1.508 and *Gluconacetobacter xylinus* (*G. xylinus*) CGMCC 1.2378 were obtained from the China General Microbiological Culture Collection Center (CGMCC, Beijing, China). *Zygosaccharomyces bailii* (*Z. bailii*) CICC 32677, *Debaryomyces hansenii* (*D. hansenii*) CICC 1714, and *Schizosaccharomyces pombe* (*S. pombe*) CICC 1056 were obtained from the China Center of Industrial Culture Collection (CICC, Beijing, China). *Lactiplantibacillus plantarum* (*L. plantarum*) S-1 was isolated and preserved in the Biomanufacturing Laboratory of Dalian Polytechnic University.

YPD broth, MRS broth, and potato dextrose agar (PDA) were purchased from Qingdao Haibo Biotechnology Co., Ltd. (Qingdao, China). HPLC-grade acetonitrile was purchased from Spectrum Chemical Manufacturing Corp. (Shanghai, China), and trifluoroacetic acid was obtained from Macklin Biochemical Co., Ltd. (Shanghai, China). Standards used for individual phenolic analysis were purchased from Macklin Biochemical Co., Ltd. (Shanghai, China), Aladdin Biochemical Technology Co., Ltd. (Shanghai, China), Yuanye Bio-Technology Co., Ltd. (Shanghai, China), and Ronghe Pharmaceutical Technology Development Co., Ltd. (Shanghai, China).

### 2.2. Preparation of Starter Cultures

The acetic acid bacteria (*A. pasteurianus* and *G. xylinus*) were cultured in a liquid medium containing 10.0% glucose and 0.1% yeast extract at 30 °C and 200 rpm for 36 h. The yeasts (*Z. bailii*, *D. hansenii*, and *S. pombe*) were cultured in YPD broth at 30 °C and 200 rpm for 36 h. *L. plantarum* S-1 was cultivated in MRS broth at 37 °C for 12 h. After cultivation, the cells were harvested by centrifugation at 8000× *g* for 10 min and resuspended in sterile 0.85% saline for subsequent fermentation inoculation.

### 2.3. Preparation of Kombucha

Kombucha preparation was performed according to Shi et al.’s method [7]. Six sweetened substrate infusions were prepared using 1 L deionized water, 100 g sucrose, and 8 g substrate. WT, GT, YT, BT, OT, and MT were infused at 90 °C for 6 min, 85 °C for 3 min, 85 °C for 3 min, 85 °C for 40 min, 85 °C for 5 min, and 85 °C for 15 min, respectively. After cooling to room temperature (25 ± 1 °C), the cell suspensions were centrifuged, resuspended in an equal volume of tea broth, and used to prepare the synthetic microbial communities (SMC1, SMC2, and SMC3). Each SMC was inoculated into 100 mL of the corresponding substrate broth at 2 mL per flask, giving a final cell concentration of approximately 1 × 10^5^ cells/mL.

SMC1 consisted of *Z. bailii*, *D. hansenii*, and *L. plantarum*; SMC2 consisted of *Z. bailii*, *D. hansenii*, *S. pombe*, *A. pasteurianus*, and *G. xylinus*; and SMC3 consisted of *Z. bailii*, *A. pasteurianus*, and *G. xylinus*. Uninoculated tea broth was used as the blank control. The selection of strains and SMC combinations was based on preliminary laboratory screening of their fermentation performance in kombucha-related systems and was chosen to represent different microbial functional compositions [5,7].

The inoculated samples were dispensed into sterilized 100 mL conical flasks, sealed with breathable film, and fermented statically at 29 ± 1 °C for 10 d. Each treatment was prepared in triplicate. Samples from the inoculated fermentation groups were collected on day 10, whereas samples from the uninoculated control groups were collected on days 0 and 10 for subsequent analyses.

### 2.4. Determination of Viable Cell Counts and pH

Viable microbial cell counts in the fermented samples were determined according to Wang et al.’s method [5]. The samples were serially diluted with sterile 0.85% saline, and 0.2 mL of each dilution was spread onto the corresponding agar media. Yeasts, acetic acid bacteria, and *L. plantarum* were enumerated on PDA, glucose–yeast extract–calcium carbonate agar (GYCA), and MRS agar after incubation at 30 °C for 3 d, 30 °C for 3 d, and 37 °C for 24 h, respectively (three dilution levels, each plated in triplicate). The results were expressed as log CFU/mL.

The pH of the tea broth was determined using a digital pH meter (FE28-Standard, Mettler-Toledo Instruments, Shanghai, China), and each sample was measured in triplicate.

### 2.5. Determination of Glucose and Ethanol Contents

The contents of glucose and ethanol were determined using a biochemical sensor analyzer (SBA-90, Institute of Biology, Shandong Academy of Sciences, Jinan, China). For ethanol quantification, the diluted samples were adjusted to pH 7.0 using 1 mol/L NaOH, and the instrument was calibrated with a 1% ethanol standard solution. After calibration, 25 μL of the prepared fermentation broth was used for the measurement. All measurements were performed in triplicate.

### 2.6. Determination of Total Phenolics, Total Flavonoids, and Antioxidant Activities

The total phenolic content was determined according to Cardoso et al.’s method [12]. The determination was performed using the Folin–Ciocalteu colorimetric method, with gallic acid as the standard. The absorbance was measured at 760 nm, and the results were expressed as mg GAE/L.

The total flavonoid content was determined according to Vitas et al.’s method [13]. The samples or standard solutions were sequentially reacted with NaNO_2_, 10% Al (NO_3_) _3_, and 4% NaOH, with 6 min standing after each step and a final reaction time of 30 min. Rutin was used as the standard, and the results were expressed as mg RE/L.

Antioxidant activities were evaluated by ABTS radical scavenging capacity (ABTS), total reducing power, and hydroxyl radical scavenging activity (HRSA). All samples were filtered through a 0.22 μm membrane filter before analysis. The ABTS capacity was determined according to Wang et al.’s method [14]. Briefly, an ABTS solution (3.2 mmol/L) was mixed with K_2_S_2_O_8_ solution (2.5 mmol/L) at an equal volume ratio and kept in the dark for 12 h to generate ABTS radical cations. The mixture was then diluted with distilled water to an absorbance of approximately 0.70 at 734 nm. A 20 μL aliquot of sample was mixed with 150 μL of the ABTS working solution, and the absorbance was measured at 734 nm. Trolox was used as the standard, and the results were expressed as mmol Trolox/L.

The total reducing power was determined according to Shi et al.’s method [7]. The reaction mixture consisted of 1 mL of the filtered sample, 1.5 mL of a phosphate buffer (0.2 mol/L, pH 6.6), and 2.5 mL of a potassium ferricyanide solution (1%, *w*/*v*). After incubation at 50 °C for 30 min, 2.5 mL of a trichloroacetic acid solution (10%, *w*/*v*) was added, and the mixture was centrifuged at 3000 rpm for 10 min. A 2.5 mL aliquot of the supernatant was mixed with an equal volume of distilled water and 0.5 mL of an FeCl_3_ solution (0.1%, *w*/*v*), and the absorbance was measured at 700 nm. Ascorbic acid (Vc) was used as the standard, and the results were expressed as mg Vc/mL.

The HRSA was determined based on the Fenton reaction [15]. The reaction mixture consisted of 400 μL of an FeSO_4_ solution (9 mmol/L), 400 μL of a salicylic acid–ethanol solution (9 mmol/L in 4% ethanol), and 200 μL of the filtered sample. After mixing, 40 μL of a H_2_O_2_ solution (8.8 mmol/L) was added, and the reaction was carried out at 37 °C for 60 min. The absorbance was then measured at 510 nm. Vc was used as the standard, and the results were expressed as mg Vc/mL.

### 2.7. Determination of Individual Phenolic Compounds

Individual phenolic compounds were determined according to Shi et al.’s method [7]. Samples were filtered through a 0.22 μm aqueous membrane before analysis. High-performance liquid chromatography coupled with a diode array detector (HPLC-DAD; Agilent 1260 Infinity II, Agilent Technologies Inc., Santa Clara, CA, USA) was used for the analysis, and chromatographic separation was performed on a C18 column (ZORBAX SB-C18, 250 mm × 4.6 mm, 5 μm). Mobile phase A consisted of 5% acetonitrile containing 0.035% trifluoroacetic acid, and mobile phase B consisted of 50% acetonitrile containing 0.035% trifluoroacetic acid. The gradient elution program was as follows: 10% B at 0 min, 20% B at 10 min, 40% B at 20 min, 90% B at 30 min, maintained at 90% B until 40 min, and returned to 10% B at 45 min. The column temperature was set at 32 °C, the detection wavelength was 205 nm, and the injection volume was 10 μL. The compounds were identified based on the retention times of authentic standards and quantified using the external standard method.

### 2.8. Electronic Nose Analysis

The electronic nose analysis was performed using an electronic nose system (PEN33, Airsense Analytics Inc., Schwerin, Germany) according to Zong et al. with slight modifications [16]. Briefly, 2 mL of fermented sample was transferred into a headspace vial and equilibrated in a water bath at 60 °C for 20 min prior to analysis. The overall odor profile of each sample was characterized using an electronic nose equipped with an array of 10 metal oxide sensors, including W1C, W5S, W3C, W6S, W5C, W1S, W1W, W2S, W2W, and W3S, whose response signals corresponded to aromatic components and benzene, nitrogen oxides, ammonia and aromatic components, hydrides, aromatic components of short-chain alkanes, methane, inorganic sulfide, alcohols and aldehydes, organic sulfides, and long-chain alkanes. Each sample was analyzed in triplicate.

### 2.9. Electronic Tongue Analysis

The taste characteristics of the fermentation-end samples were analyzed using an electronic tongue (TS-5000Z, Insent, Atsugi, Japan). Each sample was analyzed in triplicate. Based on the sensor responses, the samples were characterized in terms of sourness, bitterness, astringency, aftertaste-bitter, aftertaste-astringent, umami, richness, and saltiness [17].

### 2.10. Sensory Evaluation

A total of 20 panelists (average age: 25 years) were recruited for the sensory evaluation of the kombucha samples collected at the end of fermentation. Before the evaluation, all panelists received a brief training session to become familiar with the sensory attributes, scoring criteria, and evaluation procedure. The evaluated attributes included color, odor, taste, and overall acceptability, and a 10-point scale was used for scoring [6] (see Table S2).

All samples were coded with random three-digit numbers and presented to the panelists in a randomized order to minimize order bias. During the evaluation, the panelists were asked to rinse their mouths with drinking water between samples. Each sample was evaluated in duplicate in two independent sessions, and the mean score was used for subsequent analysis.

### 2.11. Ethical Considerations

The sensory evaluation involved healthy adult volunteers. All panelists participated voluntarily and provided written informed consent before participation. Personal information was not collected, and all sensory data were analyzed anonymously. The experiment complied with institutional requirements on raw material safety, experimental management, informed consent, and privacy protection.

### 2.12. Comprehensive Quality Evaluation

Comprehensive quality evaluation was conducted based on antioxidant activity, overall acceptability, and ethanol content, which were used to represent functional potential, sensory acceptability, and ethanol control, respectively. The comprehensive antioxidant score was calculated as the average of the Z-score normalized mean values of hydroxyl radical scavenging activity, total reducing power, and ABTS radical scavenging capacity. Z-score normalization was applied to reduce the influence of differences in units and numerical ranges among the antioxidant assays. Overall acceptability was obtained from the sensory evaluation, and the ethanol content was obtained from the ethanol measurements. Therefore, this evaluation served as a descriptive comparative tool among the treatments, rather than a predictive or weighted quality model.

### 2.13. Statistical Analysis

All experiments were performed in triplicate, and the results were expressed as means ± SD. The statistical analysis was conducted using IBM SPSS Statistics 26.0. Differences among the groups were analyzed by one-way ANOVA followed by Tukey’s multiple comparison test, with *p* < 0.05 and *p* < 0.01 considered statistically significant and highly significant, respectively. Pearson correlation analysis was used for the phenolic metabolism-related analysis. Figures were generated using Origin 2024, the ChiPlot online platform, and Python 3.12, and the heatmap data were log-transformed before visualization.

## 3. Results and Discussion

### 3.1. Microbial Growth, Acidification Characteristics, and Carbon Metabolite Accumulation

**Figure 1:**
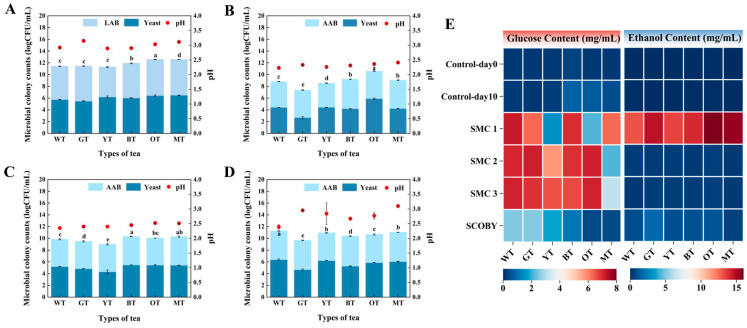
Microbial colony counts, pH values, and glucose and ethanol contents of kombucha fermented by different microbial communities across six tea substrates. (**A**) SMC1; (**B**) SMC2; (**C**) SMC3; (**D**) SCOBY; (**E**) glucose and ethanol contents. Different lowercase letters indicate significant differences among fermentation treatments within the same microbial community (*p* < 0.05). Values in panel E represent mean concentrations (mg/mL). Control indicates uninoculated tea broths. Detailed glucose and ethanol data are provided in Table S1. Abbreviations: WT, white tea; GT, green tea; YT, yellow tea; BT, black tea; OT, oolong tea; MT, mint tea.

Microbial growth and acidification are key indicators of kombucha fermentation performance. To evaluate these processes, this study compared a SCOBY and three customized microbial communities, including SMC1 (LAB—yeast consortium), SMC2 (AAB—yeast consortium), and SMC3 (AAB—yeast consortium), across six different tea substrates. As shown in Figure 1A–D, all inoculated groups maintained high viable cell counts at the 10-day fermentation endpoint. At the fermentation endpoint, the total viable counts of yeast and bacteria in the SMC1, SMC2, and SMC3 groups reached 8.95–12.60 log CFU/mL (initial inoculation: ~5.5 log CFU/mL), indicating the successful establishment of the customized microbial communities in the tea-based fermentation systems.

Accompanied by active microbial metabolism, the pH of the fermentation tea broths decreased significantly across all groups. Notably, SMC2 and SMC3, which were dominated by acetic acid bacteria (AAB), exhibited stronger acidification, with the terminal pH stabilizing between 2.2 and 2.5 (Figure 1B,C). This phenomenon is consistent with the oxidative metabolism of AAB, which convert ethanol produced by yeasts into acetic acid and other organic acids, leading to substantial acid accumulation in the fermentation system. Such acidification reflects active carbon metabolism during fermentation and contributes to maintaining a low-pH environment that supports microbial stability and product safety.

To further investigate how microbial community structure influences endpoint carbon metabolite accumulation, the accumulation of glucose (one of the primary hydrolysis products of sucrose) and ethanol (a key intermediate metabolite) was quantified in the fermentation tea broths (Figure 1E, Table S1). It is generally reported that kombucha fermentation follows a cascade conversion pathway in which sucrose is hydrolyzed by the yeast- derived invertase into fructose and glucose. These monosaccharides are subsequently converted into ethanol via alcoholic fermentation and can be further oxidized into organic acids [18].

As shown in Figure 1E and Table S1, the glucose content in the uninoculated control group (Control-day10) remained extremely low, fluctuating only between 0.15 and 0.75 mg/mL. In contrast, glucose concentrations in most fermentations inoculated with SMC1, SMC2, and SMC3 were significantly higher, reaching 6.30–7.21 mg/mL in the WT, GT, and BT substrates, which generally exceeded the levels observed in the SCOBY group (0.37–2.53 mg/mL). These results suggest that the customized microbial communities may possess an enhanced sucrose hydrolysis capacity, leading to a greater accumulation of free monosaccharides available for downstream metabolism.

Despite the similarly high monosaccharide availability, distinct carbon metabolic outcomes were observed among the different microbial communities (Figure 1E, Table S1). SMC1, composed of lactic acid bacteria (LAB) and yeast, accumulated substantial ethanol, which may be related to the absence of AAB-mediated ethanol oxidation, with final concentrations reaching 13.09–15.88 mg/mL. This level significantly exceeded the non-alcoholic threshold of 0.5% (*v*/*v*) defined by the Brazilian regulation, U.S. TTB guidance and Chinese national standards [19,20,21], which corresponds to 3.945 mg/mL based on an ethanol density of 0.789 g/mL. In contrast, the low endpoint ethanol levels (0.34–0.52 mg/mL) in the AAB-containing SMC2 and SMC3 suggest an effective metabolic coupling between yeasts and AAB. This yeast–AAB metabolic interaction highlights the critical role of community composition in shaping the final carbon metabolite accumulation and controlling endpoint ethanol levels during kombucha fermentation.

### 3.2. Phenolic Profiles and Antioxidant Activities

Polyphenol biotransformation represents a key biochemical process during kombucha fermentation. Polyphenols and their derivatives not only constitute the chemical basis of antioxidant activity, but also act as important precursors influencing astringency and overall sensory quality of the fermentation tea broth. In this context, the endpoint changes in total phenols, total flavonoids, and individual phenolic compounds were systematically investigated after 10 d of fermentation. Furthermore, by integrating hydroxyl radical scavenging activity (HRSA), total reducing power, and ABTS scavenging capacity, the relationship between phenolic transformation and antioxidant functionality was comprehensively evaluated.

#### 3.2.1. Changes in Total Phenolics and Total Flavonoids Across Tea Substrates

**Figure 2:**
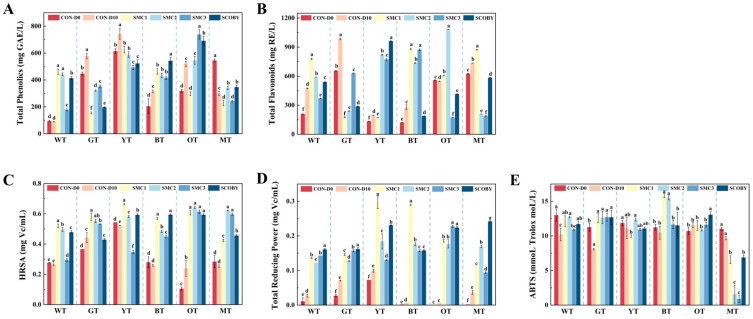
Phenolic contents and antioxidant capacities of kombucha fermented by different synthetic microbial communities (SMCs) and SCOBY across six tea substrates. (**A**) Total phenolics. (**B**) Total flavonoids. (**C**) Hydroxyl radical scavenging activity (HRSA). (**D**) Total reducing power. (**E**) ABTS radical scavenging capacity. Data are expressed as mean ± SD (*n* = 3). Different lowercase letters indicate significant differences among fermentation groups within the same tea substrate (*p* < 0.05). Abbreviations: WT, white tea; GT, green tea; YT, yellow tea; BT, black tea; OT, oolong tea; MT, mint tea.

The changes in total phenols and total flavonoids during kombucha fermentation exhibit significant substrate specificity (Figure 2A,B), indicating that the initial phenolic composition of different tea substrates was associated with the direction of metabolic transformations. Based on the structural characteristics of their dominant polyphenols, the six substrates can be broadly classified into three categories: (i) non- or lightly fermented teas (GT, WT, and YT), which are rich in catechin-type monomers such as epigallocatechin gallate (EGCG), epigallocatechin (EGC), epicatechin (EC), and epicatechin gallate (ECG) [22]; (ii) semi-fermented and fully fermented teas (OT and BT), characterized by varying degrees of oxidized polymeric polyphenols, with OT tea containing both monomeric and oxidized forms, while BT being dominated by theaflavins and thearubigins [23]; (iii) non-tea herbal-based MT, which is rich in herbal phenolic acids and flavonoid glycosides such as rosmarinic acid, neoponcirin, and chlorogenic acid, differing fundamentally from typical tea catechin systems [24].

**Figure 3:**
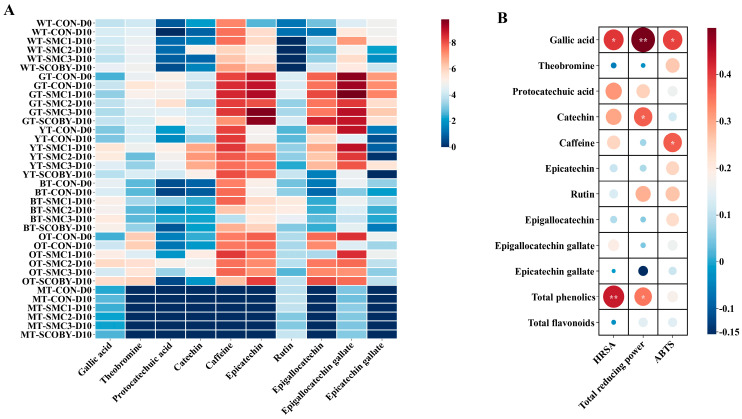
Biotransformation of monomeric phenols and their correlations with antioxidant properties. (**A**) Heat map of monomeric phenolic compounds (log-transformed data). (**B**) Pearson correlation heat map between specific phenolic compounds and antioxidant indicators. The color gradient represents Pearson’s correlation coefficient (r), and asterisks indicate significance levels (* *p* < 0.05, ** *p* < 0.01). The size of the circles is proportional to the absolute value of the correlation coefficient. Abbreviations: WT, white tea; GT, green tea; YT, yellow tea; BT, black tea; OT, oolong tea; MT, mint tea.

In catechin-rich substrates (GT, WT, and YT), although their initial compositions are similar, distinct transformation patterns were observed after fermentation. In GT, both total phenols and total flavonoids decreased significantly (decreasing to 154.90–352.06 mg GAE/L and 176.75–631.47 mg RE/L, respectively); specifically, the total flavonoids in the GT-SMC1 group dropped to 176.75 mg RE/L. This suggests catechin-type compounds are susceptible to microbial degradation and transformation under fermentation conditions, possibly resulting in changes in their detectable abundance and the accumulation of smaller phenolic compounds. However, specific transformation routes, such as ester bond hydrolysis, ring-opening reactions, or the formation of low-molecular-weight phenolic acids, require further verification. In contrast, WT exhibited a simultaneous increase in total phenols and total flavonoids, reaching up to 459.56 mg GAE/L and 777.88 mg RE/L, respectively. This may be attributed to the release of bound or physically entrapped polyphenols, as the relatively intact leaf structure of WT limits initial extractability, while fermentation-associated enzymatic and acidic conditions may facilitate their liberation (see Figure 3A) [25,26]. Moreover, YT exhibited a divergent pattern, with decreased total phenols overall in the SMC groups (dropping to 493.00–624.38 mg GAE/L) but increased total flavonoids, with the total flavonoids of the YT-SMC2 group reaching 821.28 mg RE/L, suggesting that different phenolic fractions may respond differently to fermentation, possibly involving catechin conversion and the release or formation of flavonoid-like compounds. These inferred reactions require further verification.

In contrast, semi- and fully fermented teas (OT and BT) exhibited significant increases in flavonoid content, suggesting a potential depolymerization process. Specifically, OT with its mixed composition of catechins, polymeric polyphenols, and glycosides, demonstrated strong flavonoid accumulation (e.g., 1084.30 mg RE/L in the OT-SMC2 group). This pattern may reflect the occurrence of multiple phenolic transformation processes, but further verification is required. In BT, the total flavonoids decreased in the SCOBY group but increased substantially in the SMC1 and SMC3 groups (up to ~880 mg RE/L), which was likely associated with the breakdown of polymerized polyphenols such as theaflavins and thearubigins into smaller, more detectable flavonoid compounds [27].

As a non-tea herbal substrate, MT contains phenolic acids such as rosmarinic acid rather than catechin-type polyphenols [28]. Consequently, its endpoint phenols and flavonoids exhibited no consistent trend and showed a strong dependence on microbial community composition. Total phenolic content in the fermentation groups ranged from 228.29 to 347.50 mg GAE/L, while total flavonoids in the SMC1 group reached as high as 872.75 mg RE/L, whereas in the SMC3 and SMC2 groups, they ranged from 189.50 to 208.69 mg RE/L. This suggests that differences in molecular structure may partly influence phenolic changes in MT.

#### 3.2.2. Modulation of Antioxidant Activity After Fermentation

To evaluate the impact of fermentation on antioxidant functionality, hydroxyl radical scavenging activity (HRSA), total reducing power, and ABTS scavenging capacity were measured (Figure 2C–E). Overall, distinct and assay-dependent differences in antioxidant responses were observed across substrates, reflecting the different chemical mechanisms underlying these assays [29].

HRSA and total reducing power for most substrates, which primarily depend on the electron- or hydrogen-donating capacity of compounds and are largely governed by the presence of phenolic hydroxyl groups and conjugated structures [30], were generally enhanced or maintained after fermentation in most substrates. Among them, YT exhibited the most pronounced increase, with the YT-SMC1 group exhibiting the highest HRSA and total reducing power (approximately 0.66 and 0.30 mg Vc/mL, respectively).

In GT, although fermentation significantly reduced total phenolic content, its HRSA and reducing power remained at relatively high levels (0.429–0.580 and 0.128–0.162 mg Vc/mL, respectively). This may be attributed to the fact that HRSA and total reducing power are not solely dependent on total phenolic abundance, but rather on the relative enrichment of highly active phenolic fractions during fermentation [31].

In contrast, ABTS scavenging capacity exhibited a distinct pattern. BT showed significant enhancement, with the BT-SMC1 group reaching a peak of approximately 15.8 mmol Trolox/L, suggesting that this microbial community is associated with a greater endpoint accumulation of ABTS-reactive antioxidant compounds in the BT system. In contrast, the herbal substrate MT displayed an opposite trend, where the ABTS scavenging capacity of all fermentation groups declined despite increases in HRSA and reducing power. This divergence is likely associated with its initial phenolic composition, which is dominated by phenolic acids and glycosylated flavonoids that are reported to exhibit relatively lower reactivity toward ABTS radicals.

#### 3.2.3. Metabolic Transformation of Individual Phenols and Their Relationship with Antioxidant Activity

To further clarify the specific molecular basis of the aforementioned antioxidant transformation patterns, the metabolic profiles of major monomeric phenols were analyzed. As shown in Figure 3A, significant differences were observed in the composition of monomeric phenols across different substrate fermentation systems. After fermentation, the GT, YT, and OT systems retained high levels of catechin monomers (EC, EGC and EGCG), whereas the abundance of these components was relatively low in the BT system. In contrast, the MT system showed almost no detectable levels of typical tea-derived monomeric phenols, exhibiting a distinctly different chemical composition. In addition, GT, WT, YT, BT, and OT retained relatively high levels of caffeine both before and after fermentation.

Despite substrate-specific compositions, several conserved transformation reactions were observed across fermentation systems, particularly the degradation of complex polyphenols into low-molecular-weight monomers. Specifically, the relative abundance of rutin decreased in all fermentation groups of WT and GT, suggesting possible hydrolysis or conversion of flavonoid glycosides during fermentation [32]. Concurrently, gallic acid showed a widespread accumulation trend in the fermentation groups of YT, BT, and OT, with particularly significant increases in the YT-SMC1, YT-SMC2, OT-SMC2 and OT-SCOBY groups compared to the unfermented control groups. Based on previous studies, the accumulation of this simple phenolic acid may be associated with degalloylation (i.e., the cleavage of ester bonds) of galloylated catechins such as EGCG and ECG during fermentation [33]. These inferred processes may promote the formation of low-molecular-weight phenolics with higher accessibility and chemical reactivity. Furthermore, specific transformation processes exhibited strong strain- and substrate-dependence; in the YT medium, the customized microbial communities (SMC1–3) led to a significantly higher accumulation of free catechin compared with the SCOBY group. This indicates higher transformation efficiency under specific substrate conditions.

The correlation analysis in Figure 3B further elucidated the potential links between specific phenolic components and antioxidant phenotypes. Total phenolics showed a significant positive correlation with HRSA (*p* < 0.01) and total reducing power (*p* < 0.05), suggesting that bulk phenolic content is associated with antioxidant capacity. Unlike total phenolics, total flavonoids did not show significant overall correlations with the three antioxidant indices, likely due to their structural heterogeneity and varying intrinsic activities. More importantly, gallic acid showed significant positive correlations with HRSA (*p* < 0.05), total reducing power (*p* < 0.01), and ABTS (*p* < 0.05), suggesting that gallic acid was associated with antioxidant activity in the fermented tea systems. Furthermore, specific monomers contributed to the differentiation of antioxidant phenotypes. Catechin was significantly positively correlated with total reducing power (*p* < 0.05), which is consistent with the increase in total observed reducing power in the YT fermentation group; while the positive correlation between caffeine and ABTS (*p* < 0.05) may reflect co-variation with other antioxidant-active compounds rather than a direct dominant contribution.

### 3.3. Overall Odor Profile Based on the Electronic Nose

**Figure 4:**
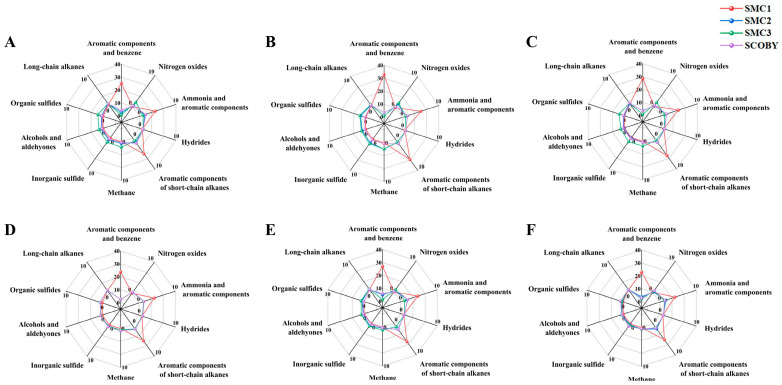
Electronic nose determination results of different tea substrates fermented Kombucha. (**A**) WT; (**B**) GT; (**C**) YT; (**D**) BT; (**E**) OT; (**F**) MT. Abbreviations: WT, white tea; GT, green tea; YT, yellow tea; BT, black tea; OT, oolong tea; MT, mint tea.

To compare the effects of different tea substrates and microbial communities on the overall aroma profile of kombucha, an e-nose analysis was performed. As shown in Figure 4, kombucha fermented from different tea substrates exhibited similar overall odor profiles, with only minor variations in sensor response intensity. In contrast, microbial community composition exerted a more significant impact on specific sensor responses. Notably, SMC1 showed the greatest deviation from other groups, while the SMC2, SMC3, and SCOBY groups displayed relatively similar response patterns. Further analysis revealed that the specificity of SMC1 was mainly associated with sensors responsive to aromatic compounds and nitrogen-containing volatiles, while the remaining sensors showed consistent trends across groups. These results suggest that microbial communities primarily influence the aroma of kombucha by modulating the response intensity of certain aroma-related volatiles [34].

### 3.4. Sensory and Taste Characteristics of Kombucha

#### 3.4.1. Taste Profile Characterized by Electronic Tongue

**Figure 5:**
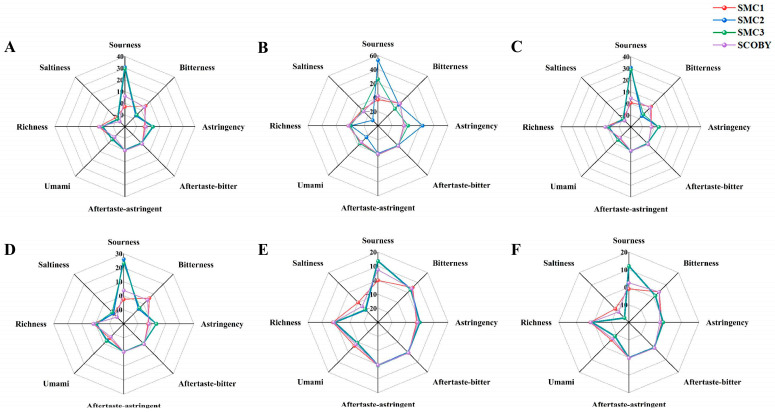
Electronic tongue determination results of different tea substrates fermented Kombucha. (**A**) WT; (**B**) GT; (**C**) YT; (**D**) BT; (**E**) OT; (**F**) MT. Abbreviations: WT, white tea; GT, green tea; YT, yellow tea; BT, black tea; OT, oolong tea; MT, mint tea.

The electronic tongue radar plots (Figure 5) reflect the differences in taste attributes across various fermentation systems. Among the evaluated dimensions, sourness emerged as the primary factor distinguishing the groups. In most tea substrates, SMC2 and SMC3 exhibited the highest sourness response values, except in GT where SMC2 exhibited an extremely high sourness response. These results are consistent with the presence of acetic acid bacteria (AAB) in both consortia, which confer strong acid-producing capacity [35]. In contrast, the SMC1 group, which lacks AAB, exhibited low sourness responses in all substrates, while the SCOBY group exhibited moderate sourness levels.

Compared with sourness, bitterness and astringency varied more among tea substrates and showed relatively smaller differences among fermentation groups [36]. Bitterness differentiation was primarily observed in WT, YT, and BT, where SMC1 and SCOBY exhibited higher response levels, whereas SMC2 and SMC3 were relatively lower. In contrast, differences among groups were minimal in OT and MT. Astringency exhibited varying patterns: SMC3 showed a higher response in WT, YT, and BT, whereas in GT, SMC2 was the most prominent. These results suggest that bitterness and astringency are more likely to be jointly influenced by the precursor compounds in the tea substrate and the extent of their microbial transformation, thereby exhibiting more complex and substrate-dependent patterns.

#### 3.4.2. Sensory Evaluation

**Figure 6:**
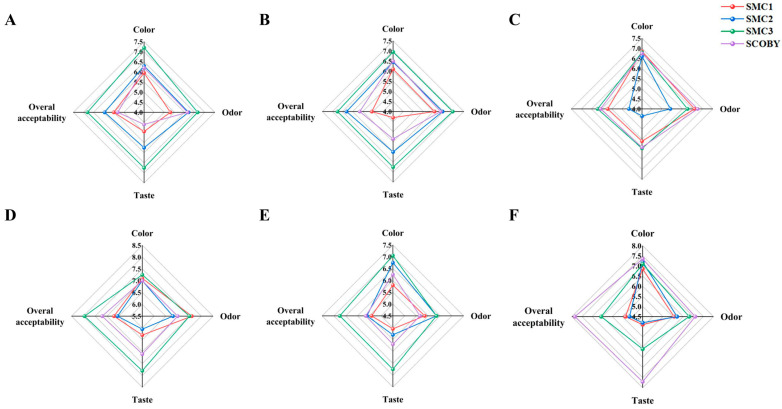
Sensory determination results of different tea substrates fermented Kombucha. (**A**) WT; (**B**) GT; (**C**) YT; (**D**) BT; (**E**) OT; (**F**) MT. Abbreviations: WT, white tea; GT, green tea; YT, yellow tea; BT, black tea; OT, oolong tea; MT, mint tea.

The sensory evaluation results (Figure 6) indicate that both tea substrates and microbial combinations influence the color, odor, taste, and overall acceptability of kombucha. Overall, color differed little among groups, whereas the other attributes showed more pronounced differences. Moreover, overall acceptability generally followed the trends in odor and taste, indicating that sensory quality was determined mainly by odor–taste harmony rather than color.

A comparison among fermentation groups further revealed distinct substrate-dependent patterns. SMC3 showed relatively higher scores for odor, taste, and overall acceptability in four tea substrates (WT, GT, BT, and OT) under the present evaluation conditions. In the YT system, SMC1, SMC3, and SCOBY showed comparable performance, while SMC2 scored significantly lower. In contrast, the MT system displayed a different trend, with SCOBY showing the highest overall score. These results suggest that the influence of different microbial communities on sensory quality was substrate-dependent [37]. Based on the endpoint sensory evaluation, SMC3 was associated with a relatively balanced sensory profile in most tea substrates, whereas SCOBY showed relatively higher acceptability in the MT system.

### 3.5. Comprehensive Evaluation of Kombucha Quality

**Figure 7:**
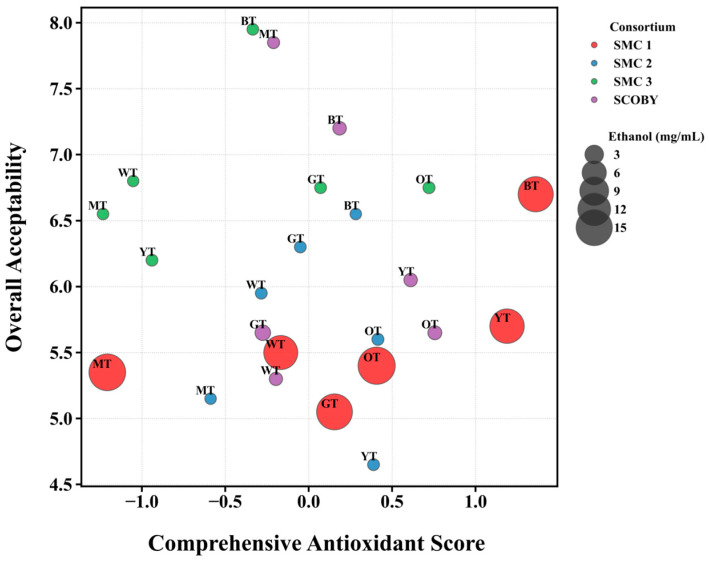
Comprehensive evaluation of kombucha samples based on comprehensive antioxidant score, overall acceptability, and ethanol content. Bubble size indicates ethanol content. Abbreviations: WT, white tea; GT, green tea; YT, yellow tea; BT, black tea; OT, oolong tea; MT, mint tea.

To comprehensively compare the overall performance of kombucha, an integrated evaluation was conducted based on antioxidant capacity, overall acceptability, and ethanol content (Figure 7). It should be noted that this integrated evaluation was intended for a comparative description of treatment performance across these quality-related attributes, rather than the establishment of a universal or weighted quality evaluation model. The results showed clear differences in the overall performance of different microbial consortia across the tea substrates.

SMC1 showed relatively higher comprehensive antioxidant scores in certain tea substrates; however, its generally higher ethanol content limited its overall performance in the integrated evaluation. In contrast, SMC2 and SMC3 maintained lower ethanol levels in most tea substrates. Notably, SMC3 also combined strong antioxidant capacity with high overall acceptability, resulting in a more balanced overall performance. Specifically, BT-SMC3 and OT-SMC3 showed better coordination among antioxidant performance, sensory acceptability, and ethanol control.

Overall, these results indicate that the quality of kombucha depends on the compatibility between tea substrates and microbial consortia. An appropriate substrate–consortium combination enables the coordinated optimization of antioxidant performance, sensory quality, and ethanol control.

## 4. Conclusions

This study showed that the tea substrate and microbial community composition jointly influenced the fermentation behavior, phenolic transformation, antioxidant activity, and sensory quality of kombucha. Microbial consortia containing acetic acid bacteria were more effective in acidification and ethanol control, whereas phenolic profiles were strongly substrate-dependent, with low-molecular-weight phenolics, especially gallic acid, being closely associated with antioxidant activity. Among the tested groups, SMC3 showed a relatively balanced overall performance across most tea substrates under the present experimental conditions. These findings provide comparative insights into the selection of tea substrates and defined microbial communities for kombucha fermentation with desirable functional and sensory characteristics. Since this study focused on endpoint characterization at Day 10, the temporal evolution and stability of these fermentation characteristics throughout the fermentation process were not evaluated and should be further investigated.

## Data Availability

The data presented in this study are available on request from the corresponding author.

## Supplementary Materials

The following supporting information can be downloaded at: https://www.mdpi.com/article/10.3390/foods15111897/s1, Table S1: Detailed data for glucose and ethanol contents shown in Figure 2; Table S2: Sensory evaluation criteria for fermentation-end kombucha samples.

## Abbreviations

The following abbreviations are used in this manuscript:

SMC1–SMC3	defined microbial communities 1–3
SCOBY	symbiotic culture of bacteria and yeast
WT	white tea
GT	green tea
YT	yellow tea
BT	black tea
OT	oolong tea
MT	mint tea
GAE	gallic acid equivalents
RE	rutin equivalents
ABTS	ABTS radical scavenging capacity
HRSA	hydroxyl radical scavenging activity
HPLC-DAD	high-performance liquid chromatography coupled with diode array detector
LAB	lactic acid bacteria
AAB	acetic acid bacteria
EGCG	epigallocatechin gallate
EGC	epigallocatechin
EC	epicatechin
ECG	epicatechin gallate
