# Supplementary material for: Defined Microbial Communities Modulate Polyphenol Transformation and Quality of Kombucha Across Different Tea Substrates
Source: Foods. 2026 May 28;15(11):1897. doi: 10.3390/foods15111897 (PMC13256360; doi:10.3390/foods15111897)
Supplement: Supplementary file 1 [file foods-15-01897-s001.zip › foods-4311590-supplementary.pdf]

Table S1: Detailed data for glucose and ethanol contents shown in Figure 2.

Types of tea	Fermentation microorganism	Glucose Content (mg/mL)	Ethanol Content (mg/mL)
WT	Control-day0	0.16±0.02[c]	0.03±0.01[d]
	Control-day10	0.20±0.01[c]	0.07±0.03[d]
	SMC 1	7.21±0.13[a]	13.09±0.19[a]
	SMC 2	7.06±0.11[a]	0.47±0.02[c]
	SMC 3	7.12±0.12[a]	0.34±0.04[c]
	SCOBY	2.52±0.42[b]	0.91±0.03[b]
GT	Control-day0	0.17±0.01[e]	0.02±0.00[c]
	Control-day10	0.15±0.03[e]	0.04±0.01[c]
	SMC 1	6.30±0.04[c]	14.69±0.45[a]
	SMC 2	7.15±0.08[a]	0.45±0.01[c]
	SMC 3	6.92±0.09[b]	0.47±0.02[c]
	SCOBY	2.53±0.10[d]	1.72±0.43[b]
YT	Control-day0	0.18±0.02[e]	0.02±0.00[d]
	Control-day10	0.23±0.01[e]	0.03±0.01[d]
	SMC 1	1.52±0.08[d]	13.42±0.28[a]
	SMC 2	5.75±0.16[b]	0.48±0.00[c]
	SMC 3	6.56±0.14[a]	0.47±0.21[c]
	SCOBY	1.83±0.16[c]	1.00±0.02[b]
BT	Control-day0	0.31±0.04[e]	0.03±0.01[d]
	Control-day10	0.75±0.06[d]	0.05±0.01[d]
	SMC 1	6.97±0.14[a]	14.04±0.15[a]
	SMC 2	6.93±0.04[a]	0.36±0.02[c]
	SMC 3	6.64±0.08[b]	0.41±0.05[c]
	SCOBY	1.08±0.04[c]	1.01±0.02[b]
OT	Control-day0	0.17±0.01[e]	0.04±0.00[d]
	Control-day10	0.63±0.11[c]	0.05±0.02[d]
	SMC 1	2.14±0.07[b]	15.88±0.18[a]
	SMC 2	7.13±0.08[a]	0.52±0.03[c]
	SMC 3	7.12±0.10[a]	0.52±0.11[c]
	SCOBY	0.37±0.03[d]	1.15±0.08[b]
MT	Control-day0	0.17±0.02[e]	0.01±0.00[c]
	Control-day10	0.45±0.04[d]	0.04±0.02[c]
	SMC 1	6.29±0.07[a]	15.43±0.30[a]
	SMC 2	2.14±0.07[c]	0.36±0.07[b]
	SMC 3	3.14±0.11[b]	0.39±0.03[b]
	SCOBY	0.40±0.03[d]	0.57±0.10[b]

Note: Data are expressed as mean ± standard deviation (n=3). Different lowercase letters in the same column indicate significant differences among fermentation treatments *($P < 0.05$)*. Abbreviations: WT: White Tea; GT: Green Tea; YT:

Yellow Tea; BT: Black Tea; OT: Oolong Tea; MT: Mint Tea; SMC: Synthetic Microbial Community; SCOBY: Symbiotic Culture of Bacteria and Yeast.

Table S2. Sensory evaluation criteria for fermentation-end kombucha samples

Attribute	Score range	Evaluation criteria
Color	8–10	Uniform and consistent color
	5–7	Slightly dark but generally acceptable color
	1–5	Dark and lusterless color
Odor	8–10	Sweet and sour aroma, with harmonious and soft odor
	5–7	Sour aroma is slightly pungent or weak, but the overall odor is relatively harmonious and soft
	1–4	Weak sweet aroma, pungent sour aroma or off-odor, and unharmonious odor
Taste	8–10	Appropriate sweet–sour balance, pure taste, and delicate, smooth mouthfeel
	5–7	Imbalanced sweet–sour ratio, slight off-flavor, and relatively delicate and smooth mouthfeel
	1–4	Overly sour or sweet, obvious off-flavor, and rough mouthfeel
Overall acceptability	8–10	Uniform and consistent color, harmonious and soft odor, appropriate sweet–sour balance, and delicate, smooth mouthfeel
	5–7	Relatively harmonious and soft odor, imbalanced sweet–sour ratio, slight off-flavor, and relatively delicate and smooth mouthfeel
	1–4	Dark and lusterless color, unharmonious odor, obvious off-flavor, and rough mouthfeel

Note: Scores were assigned on a 10-point scale, with higher scores indicating better sensory quality. The listed descriptors were used as reference criteria for evaluation.
